# Lunacy Statistics

**Published:** 1902-06-07

**Authors:** 


### LUNACY STATISTICS.

In a lecture delivered a little time ago, Dr. Claye Shaw [1] passed in review some of the changes that have occurred during the last thirty years in the treatment of lunacy. He described the many points in which marked improvement has taken place in the general system under which the great public asylums are managed, but so far as results are concerned he was far from positive as to any decided advance, and he insisted with much force upon the extremely unreliable character of many of the asylum statistics upon which the published results are founded. He said that he was inclined to think there was much truth in the view that during the past thirty years we have not made any material progress in the successful curative treatment of insanity, in other words that no more people are now discharged cured from asylums than formerly. The answer to the question when a patient is fit for discharge, depends a good deal upon the personal equation of the physician, and upon other circumstances such as the wishes of the friends to take the patient out, etc. In America the percentage of cures to admissions is not so great as it is in this country; but that is because they are much more particular before they allow anyone to leave as "cured." For the same reason they have fewer re-admitted as "relapsed" cases. In fact, said Dr. Shaw, asylum statistics are in most particulars unreliable and therefore useless.

The "results" given in asylum reports as to cures or recoveries merely show that in the opinion of a certain medical man authorised by two of the committee, such and such a person may be discharged. There is so great a pressure on the accommodation and so much interference by the friends, who exhibit great anxiety to take the patients away on the first remission of the symptoms, and there is also so much legal difficulty in keeping a patient in during the convalescent stage, especially when the time for recertification comes round, that it is to be feared that many are prematurely discharged, and if so "what becomes of the published statistics, and how can we compare the results of to-day with those of former times?" Another useless table is that giving the form of the disease in those admitted; this is merely a table of symptoms, and if the same patients were under review a week later, they would probably fall under an altogether different nomenclature. As reports are now presented, showing that so many cases of mania or so many of melancholia are admitted, "it may be truly said that they are not, as regards practical purposes, worth the paper they are written upon." This is a severe indictment, but we cannot question its truth.

[1] Lancet, May 24.

